# Improving the Quality of Postabortion Care Services in Togo Increased Uptake of Contraception

**DOI:** 10.9745/GHSP-D-16-00212

**Published:** 2016-09-28

**Authors:** Stembile Mugore, Ntapi Tchiguiri K Kassouta, Boniface Sebikali, Laurel Lundstrom, Abdulmumin Saad

**Affiliations:** a IntraHealth International, Evidence to Action Project, Washington, DC, USA; b Ministry of Health Togo, Division of Family Health, Lomé, Togo; c IntraHealth International, Chapel Hill, NC, USA; d Pathfinder International, Evidence to Action Project, Washington, DC, USA; e Pathfinder International, Evidence to Action Project, Washington, DC, USA. Now with the U.S. Agency for International Development, Washington, DC, USA

## Abstract

High-quality postabortion care (PAC) services that include family planning counseling and a full range of contraceptives at point of treatment for abortion complications have great potential to break the cycle of repeat unintended pregnancies and demand for abortions. We describe the first application of a systematic approach to quality improvement of PAC services in a West African country. This approach—IntraHealth International’s Optimizing Performance and Quality (OPQ) approach—was applied at 5 health care facilities in Togo starting in November 2014. A baseline assessment identified the following needs: reorganizing services to ensure that contraceptives are provided at point of treatment for abortion complications, before PAC clients are discharged; improving provider competencies in family planning services, including in providing long-acting reversible contraceptive implants and intrauterine devices; ensuring that contraceptive methods are available to all PAC clients free of charge; standardizing PAC registers and enhancing data collection and reporting systems; enhancing internal supervision systems at facilities and teamwork among PAC providers; and engaging PAC providers in community talks. Solutions devised and applied at the facilities during OPQ resulted in significant increases in contraceptive counseling and uptake among PAC clients: During the 5-month baseline period, 31% of PAC clients were counseled, while during the 13-month intervention period, 91% were counseled. Of all PAC clients counseled during the baseline period, 37% accepted a contraceptive, compared with 60% of those counseled during the intervention period. Oral contraceptive pills remained the most popular method during both periods, yet uptake of implants increased significantly during the intervention period—from 4% to 27% of those accepting contraceptives. This result demonstrates that the solutions applied maintained method choice while expanding access to underused long-acting reversible contraceptives. OPQ shows great potential for sustainability and scale in Togo and for application in similar contexts where the health system struggles to offer safe, high-quality, accessible PAC services.

## Abstract (precis)

The quality improvement approach applied at 5 facilities over about 1 year increased family planning counseling to postabortion clients from 31% to 91%. Of those counseled provision of a contraceptive method before discharge increased from 37% to 60%. Oral contraceptives remained the most popular method, but use of injectables and implants increased. The country-driven approach, which tended to use existing resources and minimal external support, has potential for sustainability and scale-up in Togo and application elsewhere.

*See also article by* Huber.

## INTRODUCTION

Countries in francophone West Africa have long struggled to offer women and girls safe, effective, and accessible reproductive health services, including the complete package of postabortion care (PAC) services. That package includes emergency treatment for abortion complications, family planning counseling and contraceptive methods, treatment and referral for sexually transmitted infections including HIV, and community awareness and mobilization to increase demand and acceptance of PAC services.^1^ In a region where just 17% of married women of reproductive age are using any form of contraception,^2^ more than 25% of pregnancies are unintended,^3^ and unsafe abortion services are all too common,^4^ not offering the complete package of PAC services places women and girls at undue risk of maternal death and morbidities.

In Africa in 2014, at least 9% (16,000) of maternal deaths were due to unsafe abortion.^5^ The last examination of regional statistics, in 2008, estimated that unsafe abortions account for approximately 12% of all maternal deaths in West Africa.^6^ It is widely acknowledged that availability and consistent, correct use of contraceptives to avoid pregnancy would curb maternal deaths—in part by minimizing the use of unsafe abortion services. An analysis of data from 172 countries found that in 1 year, family planning prevented an estimated 272,000 maternal deaths, achieving a 40% reduction in women dying of pregnancy-related causes.^7^ High-quality PAC services avert repeat unplanned pregnancies and the cycle of repeat abortions; they do this by providing counseling and a broad range of contraceptive services at the time and location of emergency treatment of abortion complications, and before the patient is discharged from the facility. Women who do not use contraception after an abortion are at risk of pregnancy almost immediately.^8^

It is widely acknowledged that availability and consistent, correct use of contraceptives to avoid pregnancy would curb maternal deaths—in part by minimizing the use of unsafe abortion services.

To generate evidence that would inform improvements to PAC services in West Africa, the Evidence to Action (E2A) project, funded by the United States Agency for International Development (USAID), assessed PAC services in Burkina Faso, Guinea, Senegal, and Togo from 2012 to 2013.^9^ Since 2008, the 4 countries had been participating in the Virtual Fostering Change Program^10^ to scale up best practices that would improve PAC services. The assessment’s findings were presented in 2013 at a regional meeting on PAC for francophone West African countries.^11^ Using recommendations from the assessment, country teams devised road maps for strengthening PAC services.

Since 2014, E2A, under the leadership of Togo’s Division of Family Health, has worked with the Togo country team to increase access to family planning services during PAC. This work has included expanding method choice to include long-acting reversible contraception—namely, implants and intrauterine devices (IUDs)—through a systematic approach to quality improvement at 5 health care facilities. This is the first time this systematic approach, IntraHealth International’s Optimizing Performance and Quality (OPQ) approach, has been applied and documented to improve access to high-quality PAC services in a West African country. The purpose of this article is to describe the quality improvement approach undertaken in Togo and to evaluate its effectiveness in improving contraceptive counseling and use at the health care facilities.

## METHODS

### Site Selection

The Division of Family Health within Togo’s Ministry of Health and E2A selected 5 health care facilities appropriate for applying quality improvement solutions. Selection was based on criteria that included the location of the facility, to ensure a balance in the Maritime and Plateaux regions; a substantial client load for PAC; the facility’s role as a referral site for PAC; and availability of a broad range of contraceptive methods and providers trained to offer PAC and family planning services. Two of the 5 facilities were part of E2A’s earlier assessment of PAC services in Togo.

### Baseline Assessment

E2A and the Division of Family Health conducted a baseline assessment that included site visits and a review of the 5 facilities’ organization of services, clinical records, data use and reporting, supervision systems, referral systems, equipment and supply systems, cost of services to clients, and provider competencies. The baseline assessment identified shortcomings to be addressed through the quality improvement processes (Table). These shortcomings were shared with providers, used to inform action plans for improving the services, and reassessed by E2A and supervisors during on-site supportive supervision visits.

**TABLE:** Access to and Quality of PAC Services in Togo: Baseline Assessment Findings, Quality Improvement Solutions, and Results of Applying the Solutions

Baseline Findings	Quality Improvement Solutions	Results
**Organization of services hindered access and quality:** • Separation of treatment for abortion complications (in maternity ward), FP counseling (in postnatal ward), and provision of contraceptive methods (in FP unit located in the maternal and newborn health clinic), and no formal referral system from maternity ward to FP unit. • Clients had to purchase contraceptives from the facility pharmacy, open 8 a.m. to 5 p.m. on weekdays only, and return to the maternity or FP unit for contraceptive administration or instructions on how to use the method. • Clients had to wait for services if delivery room was full, which put clients treated for septic abortion at risk of infection.	• **Facility-based quality improvement teams were oriented** on elements of successful PAC during OPQ training and supported, during on-site supervision, to reorganize services to ensure privacy during treatment and counseling, improve infection prevention practices, and ensure availability of contraceptives at point of treatment. • **Facility managers were sensitized** to the importance of postabortion FP services, which mobilized their support for creating a separate space/room for PAC treatment and FP services to be used 24 hours per day, 7 days per week.	• **All 5 clinics provide a full range of contraceptive options at point of treatment for abortion complications.** • At 3 health facilities, services were reorganized to provide FP counseling and methods around the clock at point of treatment for abortion complications, either in a separate room for PAC or in the delivery room, which is also used for emergency obstetric care. • At 2 health facilities, due to lack of space, the FP providers who provide treatment and counseling also provide contraceptives in the units to which they are allocated
**Very limited FP counseling was offered to postabortion clients, although a range of contraceptives were available at the facilities:** • At 3 facilities, no PAC clients were counseled or offered contraceptive methods. • At 2 facilities, counseling was largely reserved for the few clients who had a self-induced abortion, and contraceptive choice was limited to pills.	• **Providers were trained to improve PAC competencies**, establishing counseling for all PAC clients on the full range of contraceptives by addressing provider bias; stigma, particularly toward young unmarried clients; need for FP services regardless of whether abortion was induced or spontaneous; rights-based care; and eligibility criteria for contraceptives following emergency treatment of abortion complications.	• **Mix of contraceptive methods expanded** to include injectables, implants, and IUDs. • **Significant increases in counseling and contraceptive uptake** among all PAC clients.
**Few providers were trained to offer long-acting implants or IUDs:** • Those trained had not had a refresher training for more than 5 years in either administration of long-acting methods or emergency treatment for abortion complications.	• **Providers received competency-based trainings and follow-up support** on MVA for treatment of abortion complications and provision of contraceptives, with a focus on updating contraceptive technology competencies to address barriers to contraceptive methods, counseling skills, and provision of IUDs and implants.	• **Improved provider competencies for MVA, implants, and IUDs.** **• Significant increase in percentage of PAC clients choosing implants.**
**Four out of 5 health facilities did not have IUD kits readily available.** • Instead, providers assembled kits from other instruments in the theater and maternity ward.	• **The national quality improvement team was mobilized** to address procurement and logistics and conduct regular supportive supervision at facilities to monitor and procure stocks.	• **All facilities now have IUD kits.**
**Costs for services varied by location.** • Treatment for abortion complications cost US$25–$30, including purchasing supplies; the range of costs of contraceptives were US$0.25–0.50 for 3 cycles of oral pills, US$0.25–$1.50 for injectables, and US$4–$6 for implants and IUDs. • High costs prohibited access to more expensive contraceptives.	• **Discussions were held with facility managers and policy makers on cost of services.** • At 4 facilities, a decision was made to **offer free contraceptives to PAC clients** by using contraceptive stocks from mobile units, which were already offered for free. (At 1 facility, clients still pay for contraceptives, which continues to impede access.) • The extent to which costs were a barrier to access and influenced method choice (e.g., clients opting for less expensive contraceptives) was documented in order to influence policy on free contraceptives for clients.	• **Significant increase in uptake of contraceptive methods** among PAC clients.
**Health information systems were neither standardized nor effective:** • PAC registers were not standardized. • Data quality was generally poor—with multiple data entry points where PAC clients received services, and clients who were referred to the FP unit for contraceptives or who returned for their 7-day check-ups were not tracked. • Data were not being used for decision making.	• **PAC registers were standardized** by adapting the register from the PAC Global Resource Center for use in Togo. • Providers were oriented to complete the registers during on-site supportive supervision. • Registers were reviewed for accuracy and consistency. • Monthly data were submitted to leadership—including facility managers and the Division of Family Health. • Continuous support for data collection and analysis was offered to providers, and progress was analyzed against the desired performance detailed in facility action plans.	• **Facilities use a standardized PAC register that tracks PAC services**, including family planning counseling and uptake of contraceptives by method. • There is a marked improvement in completeness of registers and quality of data. • Data are routinely analyzed and used to monitor progress toward set performance objectives and to update performance and quality improvement plans.
**No links existed between facility-based providers and community health workers:** • Facility-based providers were not involved in community efforts to create awareness of the dangers of unsafe abortion, importance of seeking services for bleeding during pregnancy, and merits of obtaining contraceptives during the postabortion period.	• **Providers from all 5 facilities conduct talks**, in communities, antenatal, postnatal, and child health clinics, focusing on the dangers of unsafe abortion and generating demand for FP services. • National quality improvement teams are charged with strengthening community–facility links.	• **Links between communities and facilities are improved.**

Abbreviations: FP, family planning; IUD, intrauterine device; MVA, manual vacuum aspiration; OPC, Optimizing Performance and Quality; PAC, postabortion care.

### Intervention

#### Selecting a Quality Improvement Approach

The Division of Family Health selected IntraHealth International’s OPQ^12^ approach and tools for adaptation to the Togo health system. OPQ is a cyclical process for analyzing the performance of health workers, organizations, and systems and setting up solutions to build on strengths and successes. It fosters teamwork and ownership; applies a problem-solving process to address performance gaps; and develops skills in stakeholder engagement and leadership, connecting providers at facilities with support from national, regional, and district supervisors. The Togo health system has limited capacity in both number and skills of supervisors, and the Division of Family Health selected OPQ because it can be implemented and guided by an internal team at a health care facility. The 5 facilities selected for the study had already appointed the in-charges from the maternity ward and family planning unit as internal supervisors charged with overseeing PAC services.

OPQ is a cyclical process for analyzing the performance of health workers, organizations, and systems and setting up interventions to build on strengths and successes.

**Figure:**
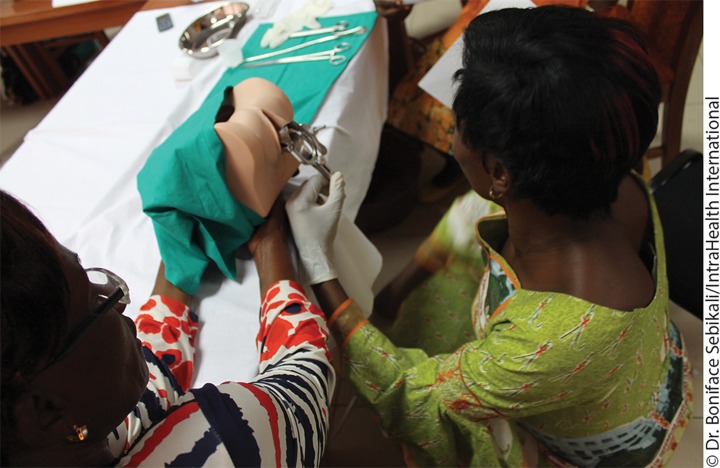
Providers practice IUD insertion on pelvic models during postabortion care training.

The Division of Family Health also formed a national quality improvement team to support the 5 health care facilities. The division national quality improvement team then developed a plan to improve access to quality family planning services during PAC, primarily by fostering teamwork and ownership of quality improvement solutions, strengthening provider competencies, addressing policy barriers, and improving how services are organized, supported, monitored, and analyzed.

#### Adapting the OPQ Methodology

To establish quality improvement measures at the 5 health care facilities, the OPQ methodology was adapted for PAC. Facilities were asked to assess their current performance (based on elements of successful postabortion family planning services as defined by a High Impact Practices in Family Planning brief^13^); define desired performance (based on the capacity of the service delivery system); identify performance gaps; and work on solutions to address the performance gaps using best practices for strengthening service delivery. Findings from the baseline assessment were integrated into OPQ to identify performance gaps, develop quality and performance objectives, and define standards against which the facilities could measure their performance.

#### Establishing a National Quality Improvement Team

The national quality improvement team included focal point persons for PAC, reproductive health, and maternal health. The team was trained to use OPQ tools and was tasked with documenting the quality improvement process, providing on-site and remote support to facility-based quality improvement teams, providing policies and guidelines on family planning and PAC, facilitating provider trainings, and creating links between community-based and facility-based services.

#### Establishing Facility-Based Quality Improvement Teams

The Division of Family Health’s plan required the in-charges of each facility’s family planning unit and maternity ward, as well as the district supervisors for family planning and reproductive health, to work together in facility-based quality improvement teams. The facility team provided leadership in conducting performance assessments, defining desired performance, identifying gaps, and implementing and monitoring quality improvement activities. The team was also tasked with obtaining resources from facility or district or regional managers to support implementation of quality improvement activities.

#### Training the Quality Improvement Teams, Phase I

The Division of Family Health, national and the facility-based quality improvement teams received a 4-day training on OPQ in November 2014. Facility teams defined their desired performance benchmarks and identified performance gaps, including their root causes. During participatory work, the teams used service data from the baseline assessment; elements of successful postabortion family planning services; and the “Ten Elements of Family Planning Success.”^14^ Using OPQ tools to explore factors that influence performance, the facility-based teams developed action plans that included solutions to close gaps and reach desired performance.

During a 4-day training, facility-based quality improvement teams defined their goals and performance benchmarks, identified performance gaps and root causes of those gaps, and developed action plans.

#### Training the PAC Service Providers, Phase II

The quality improvement action plans emphasized the need for more providers trained to provide both PAC and family planning, including long-acting contraceptive implants and IUDs. During a second training, in February–March 2015, 14 nurses and midwives from the 5 participating facilities attended a 2-week PAC training and contraceptive technology update that emphasized competency-based skills for providing implants and IUDs. The training also addressed issues such as provider bias regarding clients, including youth; the need to provide counseling and family planning methods regardless of whether the abortion was induced or spontaneous; rights-based care; eligibility criteria for family planning methods following emergency treatment of abortion complications; and recordkeeping and data use. After guided live practice to meet required practicum objectives, in June–July 2015 the trainers conducted competency-based assessments on counseling, insertion of IUDs and implants, and manual vacuum aspiration (MVA) with all 14 trained providers. These assessments resulted in certification of all participants.

#### Supporting the Facility-Based Quality Improvement Teams

In March and July 2015, the national quality improvement team and E2A offered on-site and virtual support to facility-based teams to address performance issues and barriers to implementation. Progress was assessed through observation of service delivery practices, review of registers, and interviews with providers. After each on-site support session, the national team debriefed facility managers and Division of Family Health leadership, providing feedback and soliciting needed support and resources (e.g., adequate supply of registers and contraceptives, cost waivers for PAC clients). The facility-based teams periodically updated regional and district health management teams on progress at the 5 facilities, advocating further support to improve PAC services. In August 2015, the facility-based quality improvement teams met to share preliminary results and further address performance challenges through peer-to-peer support.

### Data Collection and Analysis

We adapted the postabortion register from the PAC Global Resources Guide (http://postabortioncare.org) for use in Togo. The 5 health care facilities used the standardized register to track client indicators, including age, type of abortion complication, and method of treatment, as well as whether client was counseled, a family planning method offered and accepted, and other reproductive health services provided. To measure progress over time, monthly data were compiled and submitted to facility managers and the Division of Family Health. The facility managers and head of the Division of Family Health provided feedback and support to each facility team using information from both quantitative and qualitative monitoring. During on-site monitoring and support visits, the national quality improvement team reviewed the PAC and family planning registers for accuracy and consistency, and data were collected on the referral of clients from the maternity ward to the family planning unit or, in rare cases, to mobile units for contraceptive services. The E2A technical advisors also observed services provided, supported data collection and analysis, and analyzed progress against desired performance detailed in the facilities’ action plans. The next section describes the results of the monitoring. We plan a further evaluation to inform development of scale-up plans.

## RESULTS

### Primary Outcomes: Increased Counseling and Contraceptive Uptake

**FIGURE 1.**
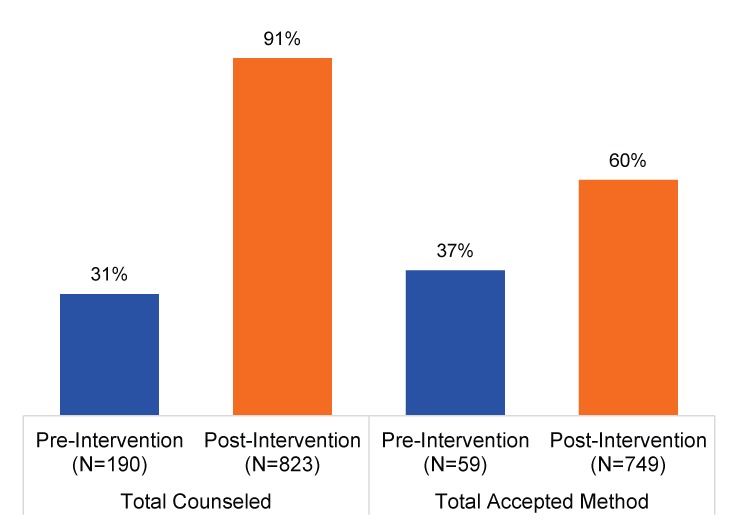
Percentage of PAC Clients Who Received Counseling and Percentage of Those Counseled Who Accepted a Contraceptive Method, Pre‐Intervention (5 Months’ Duration) and Post‐Intervention (13 Months’ Duration), 2014–2015 Abbreviation: PAC, postabortion care. Note: The pre‐intervention data include data from only 2 facilities that were offering contraceptive methods at the time, whereas the post‐intervention data include data from all 5 facilities that began offering postabortion family planning services after the Optimizing Performance and Quality training.

Since November 2014, when quality improvement processes were first initiated at the 5 health care facilities, the proportion of PAC clients who were counseled on family planning and received a contraceptive steadily increased. Overall, 91% (749/823) of women who presented for PAC services during the intervention period were counseled, and of those counseled, 60% (448/749) received a contraceptive. During the baseline period, 31% (59/190) of PAC clients were counseled, and 37% (22/59) of those received a contraceptive method (Figure 1).

Since the quality improvement processes were first initiated, the proportion of PAC clients at participating facilities who were counseled on family planning and received a contraceptive steadily increased.

**FIGURE 2.**
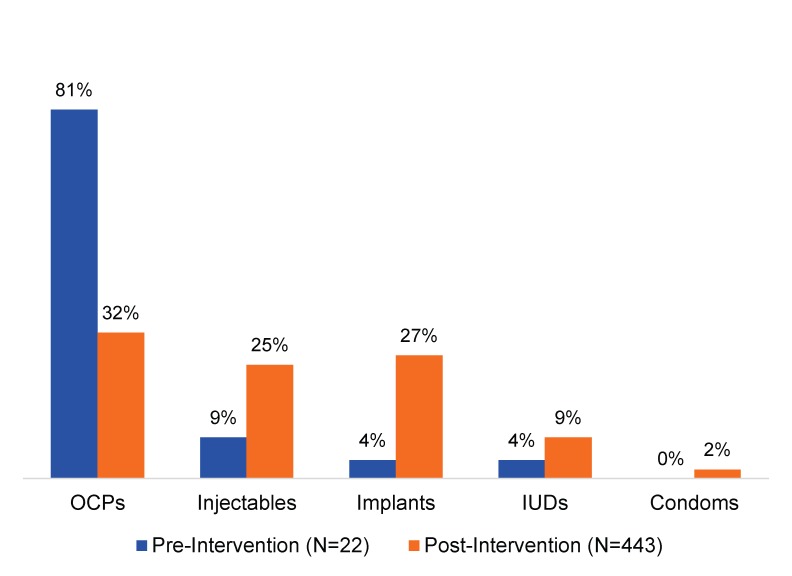
Percentage of Counseled PAC Clients Who Accepted a Contraceptive Method by Type of Method, Pre‐Intervention (5 Months’ Duration) and Post‐Intervention (13 Months’ Duration), 2014–2015 Abbreviations: IUD, intrauterine device; OCPs, oral contraceptive pills; PAC, postabortion care.

Over time, the facilities expanded the contraceptive methods that PAC clients could access to include implants, IUDs, injectables, oral contraceptive pills, and condoms. Before the intervention, 81% of the PAC clients who accepted contraceptives chose oral pills, while 4% chose implants and 4% chose IUDs. The provider trainings resulted in increased client uptake of all methods, although oral pills (32%) remained the most popular method, followed by implants (27%). Only 1 facility had IUD kits readily assembled; at that facility, from November 2014 to November 2015, 9% of clients accepting contraceptives had IUDs inserted, slightly more than double the percentage (4%) during the baseline period (Figure 2).

### Other Positive Outcomes

#### Fostering Teamwork and Ownership

Teamwork among the maternity and family planning service providers led to the removal of major barriers to offering clients contraceptives at point of treatment for abortion complications, as well as increased client access to family planning. One of the facility managers remarked:

*I like that the maternity and family planning midwives are working together and addressing their own service delivery challenges. They come with suggestions to solve problems and not just present problems.*

Provider participation in OPQ led not only to increased counseling and contraceptive uptake but also to increased overall access to family planning services, greater teamwork and engagement in community outreach among providers, and improved recordkeeping and organization of services.

#### Strengthening Provider Competencies

Having providers at all 5 facilities certified in PAC, including provision of long-acting clinical contraceptive implants and IUDs, has increased access to family planning services.

#### Improving Messages to Clients

All 5 facilities now organize talks an average of 2 to 3 times per week on PAC and healthy timing and spacing of pregnancy. These talks are directed to antenatal, postnatal, and immunization clients, including at the community level. PAC and family planning providers conduct the talks, which focus on the dangers of unsafe abortion and generating demand for family planning services.

#### Improving Service Organization

At each of the 5 health care facilities, services were reorganized so that clients could access family planning services at 3 different service delivery points: (1) point of treatment for abortion complications, (2) in the family planning unit, and (3) in a mobile unit. The majority of clients received their contraceptive methods at point of treatment, with the family planning unit the next most common delivery point. Only on rare occasions did clients receive contraceptives from mobile units.

**Point of treatment:** In 3 facilities, contraceptives are now available 24 hours per day, 7 days per week at point of treatment, either in a separate room for PAC or in the delivery room. The quality improvement teams identified a room for PAC service provision and advocated for approval and support to equip these rooms to manage PAC clients and other obstetric emergencies. One of the providers stated that “for us in our facility, maternity and family planning services are one, we no longer see them as separate.”

**Family planning unit:** In 2 facilities, due to lack of space, the providers who treat abortion complications in the maternity ward take clients to the family planning unit for counseling and contraceptives. Because the family planning unit operates only on weekdays, from 8 a.m. to 5 p.m., clients admitted for abortion complications on weekday nights are treated and then discharged the next morning so that they can access contraceptive methods before they go. On the weekends, a small stock of contraceptives is kept in the maternity ward for PAC clients.

**Mobile unit:** Mobile units in Togo provide free contraceptives, and 4 of the facilities operate a mobile unit once per month at the facility’s maternal and child health clinic, or at the regional or district health office near the facility. At these 4 facilities, clients are occasionally referred to the mobile unit for long-acting IUDs and implants. However, the main contribution of the mobile units to the quality improvement solution is in transferring contraceptive stocks to these 4 facilities so that their PAC clients can receive contraceptives for free.

#### Improving Recordkeeping and Data Use

Standardized PAC registers in all facilities, with a trained person in charge of recordkeeping, has improved data collection and quality. Data collection now includes recording clients who receive contraceptive methods during 7-day check-ups. When a client is referred to the family planning unit for contraceptives, the family planning providers record whether the client has been referred from PAC services, and the PAC providers update the PAC registers with the contraceptive method provided to the client. A review of monthly service data showed that over 90% of the PAC and family planning registers had complete information on each client, and monthly data summaries were consistent with the registers. However, use of the data at district, regional, and national levels is limited.

### Challenges Remaining

#### Strengthening Supportive Supervision at Facilities and by District Supervisors

Although the Division of Family Health provided leadership and guidance throughout the quality improvement processes, the division’s capacity to support health care facilities is limited. Continuing challenges include a shortage of staff with supportive supervision skills and their tendency to work in silos—for example, the PAC focal point person does not always involve the focal point persons for family planning and reproductive health.

#### Addressing Cost of Contraceptives

In the 4 facilities that offer mobile family planning services, district managers decided to use contraceptives from the mobile units during PAC services so that they could be provided for free. In the remaining facility participating in the intervention, clients pay for contraceptives, and cost was found to impede some clients’ acceptance and choice of methods.

#### Addressing Policies That Inhibit Access to Services

During this intervention, 4 of the health care facilities intentionally chose to use the contraceptives provided by their mobile units to show that more clients will use contraceptives when they are available for free. Discussions about how the government can ensure free contraceptives at health care facilities are ongoing. A positive outcome in terms of standards and guidelines is that the Division of Family Health adopted the training materials used for the intervention as Togo’s national PAC training curriculum.

Togo’s Division of Family Health has adopted the training materials used for this intervention as the national PAC training curriculum.

## DISCUSSION

PAC clients are an underserved, vulnerable group of women. Country ownership of quality improvement and a country-led OPQ approach had a positive effect on these women’s access to family planning counseling and choice of contraceptive methods. Our results show that applying an evidence-based, participatory approach to quality improvement has the potential to increase the accessibility and quality of services in a short time. The improvements to service delivery, which were largely driven by managers and providers at the 5 health care facilities and tended to use existing resources, required minimal external support. The feasibility of these improvements encouraged government commitment. Involving both national government and regional and district health officials from the beginning ensured their buy-in and investment in improving the quality of services and honoring PAC clients’ right to high-quality family planning services, including a full range of contraceptive methods. This approach could be adapted and applied in similar contexts, particularly in other West African countries, where PAC programs face similar challenges.

Applying an evidence-based, participatory approach to quality improvement has the potential to increase the accessibility and quality of services in a short time.

The facilities’ quality improvement solutions led to an expanded mix of contraceptive methods offered to PAC clients and to significant increases in contraceptive uptake. However, clients’ most commonly chosen method—oral contraceptive pills—remained the same during the baseline and intervention periods. This may be due to the fact that when midwives have a heavy workload and the family planning unit is not operating, oral contraceptive pills are the easiest method to provide. Togo’s Ministry of Health is considering a task-sharing strategy that would enable delivery assistants to provide implants. This is a strategy that implementing partners who support PAC and family planning in Togo should monitor closely to determine whether it makes a significant difference in which contraceptives women choose.

Ongoing challenges to PAC contraceptive uptake include a shortage of supervisory staff and their tendency to work in silos.

Many abortion clients are young people. Despite this fact, many service providers remain averse to encouraging young people to space or delay pregnancy, particularly if a young client has just experienced her first pregnancy. E2A is currently in the process of adding a youth-friendly PAC component to Togo’s national training curriculum. The revised curriculum will address provider bias and stigma to improve young people’s access to quality, client-focused PAC services that offer the full contraceptive method mix, including long-acting contraceptive implants and IUDs. The Government of Togo will need to remain focused on ensuring that young people have access to family planning services during PAC in order to honor their reproductive rights and choices and to truly have an impact on the health of women and girls in the country.

Many service providers remain averse to encouraging young people to space or delay pregnancy.

During this study’s baseline assessment, stock-outs of contraceptive methods were not identified as an issue that needed to be addressed through the quality improvement solutions. However, 4 of the 5 facilities had limited capacity to offer full method choice because they did not have IUD kits readily assembled. Additionally, only 2 facilities had functional MVA kits. To manage incomplete abortion, providers who did not have an MVA kit used manual digital removal of retained products from conception or referred clients to doctors for dilation and curettage. After training additional providers in the 5 facilities and equipping each facility with, on average, 5 MVA kits provided by Ipas, MVA became more common than manual digital removal. To ensure quality care going forward, it will be essential for all facilities that offer PAC to have the necessary equipment and commodities available at time of service.

## CONCLUSION

The quality improvement approach described in this article involved national stakeholders, regional and district health officials, and health facility managers. It was found to enable joint problem solving, and has given Togo’s public health system the impetus to sustain and scale up high-quality PAC services. The Division of Family Health has expressed a commitment to continue supporting the quality improvement teams and is now looking at how to apply lessons learned to scale up OPQ to other health care facilities and institutionalize the approach and relevant tools in routine supervision. Due to limited capacity, the Division of Family Health will require continued support from willing partners and bilateral projects to scale up OPQ for PAC and to apply OPQ to improve the quality of family planning services. It is also likely that the package of solutions will need to be simplified, given the division’s limited skills in training providers and supervisors on OPQ and providing on-site support. The division is also challenged by resource limitations, a shortage of midwives, and inadequate infrastructure and equipment for reorganization of services. Thus, sustaining the positive changes realized through OPQ and expanding the solutions to additional health care facilities in Togo will require continued ownership, support, and political will, through efforts including the following:

The Optimizing Performance and Quality approach was found to enable joint problem solving and has given Togo’s public health system the impetus to sustain and scale up high-quality PAC services.

- Building the capacity of PAC providers, including in application of OPQ
- Providing on-site supportive supervision, especially as staff turn over from their current roles
- Training new quality improvement teams as staff move or retire
- Addressing both the cost of PAC services, including contraceptives, and commodity security through shifts in policy

## Notes

**Peer Reviewed**

## References

[1] United States Agency for International Development (USAID), Postabortion Care Working Group Post abortion care strategy. Washington (DC): USAID; 2004 Available from: http://www.postabortioncare.org/sites/pac/files/USAID_PAC_Strategy.pdf

[2] United Nations (UN), Department of Economic and Social Affairs, Population Division Trends in contraceptive use worldwide 2015. New York: UN; 2015 Available from: http://www.un.org/en/development/desa/population/publications/pdf/family/trendsContraceptiveUse2015Report.pdf

[3] Sedgh G Singh S Hussain R. Intended and unintended pregnancies worldwide in 2012 and recent trends. Stud Fam Plann. 2014;45(3):301–314. 10.1111/j.1728-4465.2014.00393.x. 25207494 PMC4727534

[4] Singh S Maddow-Zimet I. Facility-based treatment for medical complications resulting from unsafe pregnancy termination in the developing world, 2012: a review of evidence from 26 countries. BJOG. 2016;123(9):1489–1498. 10.1111/1471-0528.13552. 26287503 PMC4767687

[5] Singh S Darroch J Ashford L Adding it up: the costs and benefits of investing in sexual and reproductive health. New York: Guttmacher Institute; 2014 Available from: https://www.guttmacher.org/sites/default/files/report_pdf/addingitup2014.pdf

[6] World Health Organization (WHO) Unsafe abortion: global and regional estimates of incidence of unsafe abortion and associated mortality in 2008. Sixth edition Geneva; WHO; 2011 Available from: http://apps.who.int/iris/bitstream/10665/44529/1/9789241501118_eng.pdf

[7] Ahmed S Li Q Liu L Tsui AO. Maternal deaths averted by contraceptive use: an analysis of 172 countries. Lancet. 2012;380(9837):111–125. 10.1016/S0140-6736(12)60478-4. 22784531

[8] International Federation of Gynecology and Obstetrics; International Confederation of Midwives; International Council of Nurses; United States Agency for International Development; White Ribbon Alliance; Department for International Development Post abortion family planning: a key component of post abortion care. Washington (DC); 2013 Available from: http://www.figo.org/sites/default/files/uploads/project-publications/PAC-FP-Joint-Statement-November2013-final_printquality.pdf

[9] Fikree F Mugore S Forrester H Postabortion care: assessment of postabortion care services in four francophone West Africa countries. Washington (DC): Evidence to Action Project; 2014 Available from: http://www.e2aproject.org/publications-tools/pdfs/pac-fp-assessment-report.pdf

[10] Management Sciences for Health (MSH). The virtual fostering change program. Cambridge (MA): MSH; 2010 Available from: https://www.msh.org/resources/virtual-fostering-change-program

[11] Evidence to Action Project (E2A) Report of the second regional francophone West Africa Postabortion Care Meeting: strengthening postabortion family planning. Washington (DC): E2A; 2014 Available from: http://www.e2aproject.org/publications-tools/pdfs/report-of-the-pac-workshop-oct-2013.pdf

[12] IntraHealth International Optimizing performance and quality. Chapel Hill (NC): IntraHealth International; 2013 Available from: http://www.intrahealth.org/files/media/optimizing-performance-and-quality/OPQ_FINAL.pdf

[13] High Impact Practices in Family Planning (HIP) Postabortion family planning: strengthening the family planning component of postabortion care. Washington (DC): United States Agency for International Development; 2012 Available from: http://www.fphighimpactpractices.org/resources/postabortion-family-planning-strengthening-family-planning-component-postabortion-care

[14] Johns Hopkins Center for Communication Programs (CCP) Health, Population, Nutrition eToolkit for Field Workers [Internet]. Baltimore (MD); CCP, Knowledge for Health Project; [last updated 2015 Nov 22; cited 2016 Mar 1] Available from: https://www.k4health.org/resources/health-population-nutrition-etoolkit-field-workers

